# Evaluation of a sample-to-answer real-time PCR assay for enterovirus detection in cerebrospinal fluid

**DOI:** 10.1128/jcm.00864-25

**Published:** 2025-10-21

**Authors:** Grace Perkins, Jennifer Swink, Kevin Wade, Lori Hughes, Bijal A. Parikh, Neil Anderson

**Affiliations:** 1 Department of Pathology and Immunology, Washington University School of Medicine 12275, St. Louis, Missouri, USA; 2 Barnes-Jewish Hospital 21737, St. Louis, Missouri, USA; 3 Department of Pathology, Case Western Reserve School of Medicine 12304, Cleveland, Ohio, USA; Mayo Clinic Minnesota, Rochester, Minnesota, USA

**Keywords:** virology, pcr, enterovirus

## Abstract

Enteroviruses frequently cause aseptic meningitis, necessitating differentiation from bacterial meningitis to avoid unnecessary antimicrobial treatment and improve patient management. Polymerase chain reaction (PCR) remains the gold standard for enterovirus detection in cerebrospinal fluid (CSF) due to its high sensitivity and rapid turnaround time. The discontinuation of the Cepheid Xpert EV assay has created a gap in diagnostics. This study aimed to optimize two laboratory-developed real-time PCR assays using Diasorin direct amplification disc (DAD) and universal disc (UD) methods compared to the previously standard Cepheid Xpert EV assay. A total of 87 clinical CSF specimens were tested to assess sensitivity, specificity, and overall performance. There was no significant difference between the performance of the three assays compared to standard of care results. The Xpert EV and DAD methods had a 79.6% positive agreement, while the UD method had an 86.4% positive agreement. All methods achieved 100% negative agreement. Specificity testing against non-enteroviral pathogens confirmed no cross-reactivity. The estimated limit of detection was 100 copies/mL for Xpert EV, 250 copies/mL for UD, and 1,000 copies/mL for DAD, though clinical sensitivity remained high. Blood contamination affected the DAD assay but not the UD method due to its RNA extraction step. This study demonstrates the potential of DAD and UD methods as viable alternatives for rapid enterovirus detection in CSF, essential for patient management following the discontinuation of the Xpert EV assay. Further validation and comparison are needed for broader clinical applications.

**IMPORTANCE:**

Rapid and accurate detection of enteroviruses in cerebrospinal fluid is crucial for patient management in cases of aseptic meningitis, especially following the discontinuation of the Cepheid Xpert EV assay. This study evaluates two laboratory-developed real-time PCR assays utilizing Diasorin direct amplification disc and universal disc methods, demonstrating their potential as viable alternatives for enterovirus detection with high sensitivity and specificity.

## INTRODUCTION

Enteroviral infections are common and generally asymptomatic or self-limiting (1). Nevertheless, enterovirus is the leading cause of aseptic meningitis, responsible for 80%–92% of cases globally (2). Distinguishing aseptic meningitis from the more lethal bacterial meningitis based solely on symptoms remains challenging. While treatment for aseptic meningitis is primarily supportive, bacterial meningitis requires targeted antimicrobial therapy and has a mortality rate of up to 50% (3). Thus, a rapid diagnostic test for enterovirus in cerebrospinal fluid (CSF) is critical. A timely positive CSF enteroviral result can prevent needless antibiotic exposure in patients, as well as promote more swift symptomatic management and triage (2).

Polymerase chain reaction (PCR) is the gold standard for the detection of enterovirus in CSF specimens due to its advantages of fast turnaround time and high sensitivity compared to viral culture (4, 5). Currently, there are few Food and Drug Administration (FDA)-cleared enterovirus diagnostic PCRs for CSF specimens. The BioFire FilmArray Meningitis/Encephalitis panel and QIAstat-DX Meningitis/Encephalitis panels are multiplex PCRs and array diagnostic methods that assess for common bacterial, viral, and fungal pathogens of the central nervous system and yield results in approximately 1 hour. The FilmArray Meningitis/Encephalitis panel for enterovirus detection has a reported sensitivity of 89% and specificity of 100% (6). The QIAstat-DX ME panel has variable sensitivity based on the enteroviral strain but a reported sensitivity of contrived specimens of 88%–99% (7, 8). The Cepheid Xpert EV assay is the only FDA-cleared assay targeting solely enterovirus. The assay is a real-time RT-PCR assay that results in approximately 2.5 hours with a reported sensitivity of 96% and specificity of 99% (9). Unfortunately, this assay has been discontinued by the manufacturer as of October 2023. Given the limited diagnostic options for enteroviral meningitis, we describe the evaluation of a laboratory-developed sample-to-answer real-time PCR assay utilizing commercially available analyte-specific reagents for the detection of enterovirus from CSF specimens.

## MATERIALS AND METHODS

### Specimens

This study was approved by the Washington University Institutional Review Board (ID#202312029). A total of 87 previously analyzed CSF specimens submitted for routine clinical testing at Barnes Jewish Hospital Molecular Infectious Disease Laboratory and Saint Louis Children’s Hospital Microbiology Laboratory were utilized in this study. Specimens ranged in collection dates from September 2004 to July 2023 and were stored at –80°C after initial clinical analysis. Inclusion criteria required at least 1 mL of CSF specimen to allow all necessary testing to be performed. A total of 44 of the specimens were previously enterovirus-positive by standard-of-care molecular diagnostic methods, with the other 43 specimens being previously enterovirus negative by standard-of-care molecular diagnostic methods. From 2004 to 2008, the standard-of-care molecular diagnostic method at Barnes Jewish Hospital and Saint Louis Children’s Hospital was a laboratory-developed test utilizing a Qiagen One-Step RT-PCR kit. The method utilized from 2008 to 2024 was the Cepheid Xpert EV RT-PCR assay.

### DAD protocol optimization

Four PCR protocols were tested to determine the best performance using the Diasorin 8-well direct amplification disc (DAD) (Diasorin, Cypress, California). The DAD is an 8-well, sample-to-answer PCR amplification disc that does not require RNA extraction prior to use. The four protocols were trialed with and without RNasin Ribonuclease Inhibitor (Catalog No: N2615; Promega), as well as with or without a filtration step to achieve maximum performance. Analyte-specific reagents for this assay were obtained from Diasorin. The Diasorin Enterovirus primer pair (MOL9020) is comprised of a Scorpion primer/probe that targets a conserved 5′ non-coding region. The primer pair targets Enterovirus serotypes 68, 70, and 71, as well as other Coxsackieviruses and Echoviruses. All reactions were prepared immediately before amplification as master mixes containing all reaction components: (i) 1 µL of Enterovirus primer pair, (ii) 20 µL TA master mix (MOL9070), (iii) 1 µL Reverse Transcriptase (MOL9018), (iv) 1 µL Simplexa Extraction and Amplification Control (SEAC) RNA primer pair (MOL9200), (v) 3 µL SEAC RNA template (MOL9200), and (vi) 23 µL water. Fifty microliters of sample or control was added to the “SAMPLE” well, and 50 µL of reaction mix was added to the “R” well.

Dilutions of control material were made by diluting commercially available inactivated Coxsackievirus A9 (Exact Diagnostics) in CSF matrix at concentrations of 100,000, 10,000, 5,000, 1,000, 500, 250, 125, and 62.5 copies/mL, based on the value provided by the manufacturer. Fifty microliters of diluted control material was used per reaction. RNasin was added to the PCR master mix at 1 µL per reaction. An Amicon Ultra 0.5 mL filter tube (Catalog No: UFC501096) was utilized, with filtration RPM at 14,000 g for 2 minutes, as per the package insert. PCR amplification was performed using the Diasorin LIAISON MDX instrument. All reactions were prepared immediately prior to amplification. Target and internal control fluorescence thresholds were 2,000 mean fluorescence intensity units. Data collection and analysis were performed with the LIAISON MDX Studio software. The protocol definitions and results can be found in the supplemental materials as Table S1.

Protocols were evaluated for sensitivity by comparing cycle thresholds for dilutions of control material. The dilutions evaluated and the results can be seen in the supplemental materials as Table S2. Protocol 2 with RNasin was chosen given its balance of sensitivity and favorable cycling parameters (Ct values less than 40). This protocol will be referred to from here as the “DAD method” and will be compared to the 96-well format Diasorin universal disc (UD) as well as the Cepheid Xpert EV performance.

### Diasorin UD protocol

The Diasorin UD protocol utilizes the consumable 96-well UD (Diasorin) for PCR amplification, which requires an additional RNA extraction step. Before extraction, 400 µL of specimen or control was spiked with 15 µL of SEAC RNA template (MOL9200). The SEAC RNA acted as an internal control for the extraction and amplification of the reaction. RNA extraction was performed on the bioMerieux NucliSENS easyMAG (bioMerieux, Maryc l’Etoile, France). Five microliters of extracted CSF samples or controls was used. The remaining reaction components totaled 5 µL for a final reaction volume of 10 µL. All reactions were prepared immediately prior to amplification as master mixes containing all reaction components: (i) 0.2 µL Enterovirus primer pair (MOL9020), (ii) 4 µL TA master mix (MOL9070), (iii) 0.5 µL Reverse Transcriptase (MOL9018), (iv) 0.2 µL SEAC RNA primer pair (MOL9200), and (v) 0.1 µL water PCR amplification was performed using the Diasorin LIAISON MDX instrument, and data collection and analysis were performed with the LIAISON MDX Studio software.

### Cepheid Xpert EV protocol

The Cepheid Xpert EV assay is a sample-to-answer reverse-transcription PCR (RT-PCR) assay for the detection of enteroviral RNA in CSF specimens. The protocol was followed as per manufacturer instructions on the GeneXpert Dx system.

### Comparative accuracy assessment

The DAD method, the UD method, and the Xpert EV assays were performed on 87 previously tested clinical CSF specimens. The performance of each tested method was compared to the clinical standard-of-care reported result. Positive percent agreement was calculated as the proportion of positive standard of care results that also tested positive by each of the three tested methods. Negative percent agreement was calculated as the proportion of negative standard of care results that also tested negative by each of the three methods. Overall percent agreement for each method was also calculated. Confidence intervals were calculated via bootstrapping. Cohen’s Kappa was calculated and interpreted using Landis and Koch’s classification of agreement. Additionally, the result agreement between each of the three tested assays was determined to help compare performance.

### Analytical specificity assessment

The analytical specificity of both DAD and UD assays was assessed by utilizing residual clinical CSF samples that had previously tested positive for non-enteroviral pathogens. Additionally, control samples that contained multiple pathogens were tested. The pathogens tested were herpes simplex virus 1 (HSV-1), HSV-2, Human herpesvirus 6, Varicella zoster virus (VZV), adenovirus, cytomegalovirus, *Mycoplasma pneumoniae*, Parvovirus B19, and Parechovirus.

### Comparative analytical sensitivity assessment/limit of detection

Serial dilutions of control material (inactivated Coxsackievirus A9, Exact Diagnostics) at 5,000, 1,000, 500, 250, and 100 copies/mL were run in triplicate using the Xpert EV, DAD, and UD assays. The performance of the DAD assay for enterovirus D68, which the Xpert EV assay detects less efficiently due to a higher limit of detection for this serotype, was compared to that of Xpert EV (7). Dilutions of Fermon enterovirus D68 RNA at 5,000, 1,000, 500, 250, 100, 50, and 25 TCID50/mL using previously enterovirus-negative patient CSF were run in triplicate on both Xpert EV and DAD assays. The estimated limit of detection for each assay was determined as the concentration at which three of three (100%) of replicates were detected.

### Inhibition assessment

To ensure the assay performance with the most likely inhibitory substance encountered in CSF, we performed both DAD and UD assays with a dilution of CSF and EDTA blood. CSF specimens that tested previously positive for enterovirus were pooled to make a single pooled specimen. The pooled specimen was run “neat” in addition to dilutions A, B, and C. The total volume of each dilution was 500 µL. Dilution A was made with 25 µL EDTA blood in 475 µL of the pooled specimen (dilution factor 1:20). Dilution B was made with 50 µL EDTA blood in 450 µL pooled specimen (dilution factor 1:10). Dilution C was made with 100 µL EDTA blood in a 400 µL pooled specimen (dilution factor 1:5).

## RESULTS

### Demographic results

Demographic information for patients from whom clinical specimens were utilized is listed in Table 1. Due to the age of specimens, some records predate the currently used electronic medical record, and thus, certain elements were unable to be recovered (listed as “unknown”). Patient ages were stratified into three groups: 0 to <2 years, 2 to <18 years, and >18 years. The Freeman-Halton extension of the Fisher exact test was utilized to analyze this data. There was a statistically significant difference (*P* < 0.001) between age groups, suggesting that the positive cohort was more likely to be composed of very young children (<2 years of age). We used the Fisher’s exact test to evaluate the differences in sex composition between the two groups, with statistical significance set at *P* < 0.05. There was not a significant difference between the positive and negative cohorts’ sex composition. For the remainder of the data points, we utilized the Wilcoxon rank sum test to compare the differences between the positive and negative cohorts. The significant differences between the positive and negative cohorts are seen in the nucleated cell count and whether additional viral testing of CSF was performed. The positive cohort had higher nucleated cell counts in CSF than the negative cohort. The negative cohort was more likely to have concurrent viral testing for HSV, VZV, and Parechovirus in CSF. These findings are to be expected, as a negative enterovirus test for a viral meningitis clinical picture would prompt clinicians to perform other viral testing of CSF.

**TABLE 1:** Demographic characteristics of both cohorts^*c*^

Characteristic	Negative *N* = 43^*a*^	Positive *N* = 44^*a*^	*P*-value^*b*^
Age (pediatric, <18 yrs)			<0.001
0 to <2 years	4	20	
2 to <18 years	15	19	
18+ years	22	5	
Unknown	2	NA^*d*^	
Sex			0.39
Female	21 (49%)	26 (59%)	
Male	22 (51%)	18 (41%)	
CSF glucose (mg/dL)	59 (51, 69)	56 (52, 62)	0.4
Unknown	8	13	
CSF protein (mg/dL)	42 (27, 70)	44 (29, 67)	>0.9
Unknown	9	14	
CSF lymphocytes	75 (24, 93)	49 (22, 75)	0.3
Unknown	30	19	
CSF nucleated cells	5 (4, 9)	11 (5, 50)	0.018
Unknown	8	11	
Concurrent HSV testing	31 (82%)	13 (38%)	<0.001
Unknown	5	10	
Concurrent VZV testing	16 (42%)	2 (5.9%)	<0.001
Unknown	5	10	
Concurrent Parechovirus testing	18 (47%)	6 (18%)	0.008
Unknown	5	10	

^*a*^ Median (Q1,Q3); *n*(%).

^*b*^ Wilcoxon rank sum test; Freeman-Halton test, Fisher exact test.

^*c*^ Specimen collection dates for positive cohort: 2004–2008 (15 specimens), 2009–2013 (18 specimens), 2014–2018 (6 specimens), 2019–2023 (5 specimens). Specimen collection dates for negative cohort: 2004–2008 (0 specimens), 2009–2013 (0 specimens), 2014–2018 (17 specimens), 2019–2023 (26 specimens).

^*d*^ NA, not applicable.

### DAD protocol optimization

The results of protocol optimization are depicted in Table S2. Protocol two with RNasin was selected as the most sensitive protocol with Ct values below 40.

### Comparative accuracy

**Fig 1:**
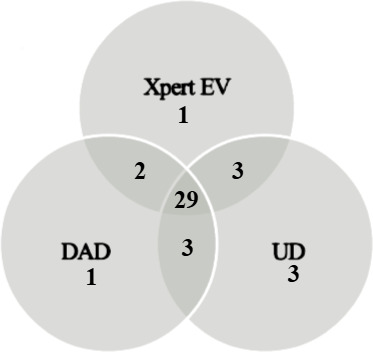
Positive result agreement of the three tested methods.

The accuracy results for all three tested methods are shown in Table 2. Utilizing the previously obtained standard of care testing as the gold standard, there was no significant difference between the performance of the three assays. The Xpert EV and DAD assays both identified 35 of 44 previous positive specimens, with a positive percent agreement of 79.6% (95% confidence interval [CI] 67.5%–90.7%) and Cohen’s Kappa of 0.794. The UD 96-well assay correctly identified 38 of 44 positive specimens, with a positive percent agreement of 86.4% (95% CI 75.6%–95.5%) and Cohen’s Kappa of 0.862. All three assays identified 43 of 43 previously negative specimens as negative, equating to a negative percent agreement of 100% for all three assays. The overall percent agreement for both the Xpert EV and the DAD assays was 89.7% (95% CI 83.9%–95.4%). The overall percent agreement for the UD assay was slightly higher at 93.1% (95% CI 87.4%–97.7%). Invalid results were not observed in any of the three tested assays. The positive result agreement between each of the three tested assays is shown in Fig. 1. Twenty-nine of 44 (66.0%) positive specimens tested positive for enterovirus by all three methods. Thirteen of 44 (30.0%) positive specimens tested positive by one or multiple methods, but not by all three methods. Two of 44 (5.0%) previously positive specimens tested negative by all three methods. Utilizing a two out of three composite reference standard, the DAD showed 91.9% sensitivity and 98% specificity, the UD had 94.6% sensitivity and 94% specificity, and the Xpert EV demonstrated 91.9% sensitivity and 98% specificity. Discordant results show Ct values ranging from 32.2 to 37.9 (Xpert EV), 39.4–41.1 (DAD), and 37.1–38.9 (UD). These findings suggest that the discordance may be due to low viral load in these specimens, given higher Ct values.

**TABLE 2:** Comparative accuracy of the three tested methods^*a*^

Accuracy of Xpert EV		
	Standard of care molecular testing result	
Xpert EV result	Positive	Negative
Positive	35	0
Negative	9	43
*PPA: 79.6% (95% CI: 67.5%–90.7%), NPA: 100%, OPA: 89.7% (95% CI: 84.9%–95.4%*)		

Accuracy of UD^*c*^		
	Standard of care molecular testing result	
UD result	Positive	Negative
Positive	38	0
Negative	6	43
*PPA: 86.4% (95% CI:75.6%–95.5%), NPA: 100%, OPA: 93.1% (95% CI:87.3%–97.7%*)		

Accuracy of DAD^*b*^		
	Standard of care molecular testing result	
DAD result	Positive	Negative
Positive	35	0
Negative	9	43
*PPA: 79.6% (95% CI:67.5%–90.7%), NPA: 100%, OPA: 89.7% (95% CI: 84.9%–95.4%*)		

^*a*^ Key: PPA (positive percent agreement), NPA (negative percent agreement), OPA (overall percent agreement).

^*b*^ DAD, direct amplificiation disc.

^*c*^ UD, universal disc.

### Comparative analytical specificity

None of the tested pathogens elicited a positive result on the DAD and UD assays, which suggests high specificity for enteroviral detection among other common CSF pathogens.

### Comparative analytical sensitivity assessment/ limit of detection

The results of the estimated limit of detection studies are depicted in Table 3. Of the three methods tested, the Xpert EV had the lowest estimated limit of detection at 100 copies/mL. The UD method had the second-lowest estimated limit of detection, estimated to be 250 copies/mL. The DAD method had an estimated limit of detection of 1,000 copies/mL. Although this suggests a difference in analytical sensitivity for the tested control material, the difference in clinical sensitivity is likely minimal, given the results of patient sample testing. The positive percent agreements between the standard of care Xpert EV method and the proposed DAD and UD methods were very similar, with the UD method having the greatest positive percent agreement at 86.4%. The negative percent agreement for all three methods was identical. The results of the D68 comparative analysis are depicted in Table 4. The DAD assay had a lower estimated limit of detection of 25 TCID50/mL, while the Xpert EV assay had an estimated limit of detection of 100 TCID50/mL. These results suggest that the DAD assay is more sensitive than the Xpert EV assay for the detection of this serotype.

**TABLE 3:** Estimated limit of detection of the three tested methods for enterovirus

	Enterovirus detected		
Nucleic acid concentration (copies/mL)	Xpert EV	DAD^*b*^	UD^*c*^
5,000	3/3	3/3	3/3
1,000	3/3	3/3	3/3
500	3/3	1/3	3/3
250	3/3	0/3	3/3
100	3/3	0/3	1/3
Estimated LoD^*a*^ (copies/mL)	100	1,000	250

^*a*^ LoD, limit of detection.

^*b*^ DAD, direct amplification disc.

^*c*^ UD, univeral disc.

**TABLE 4:** Estimated limit of detection of DAD and UD methods for enterovirus serotype D68

	Enterovirus detected	
Viral dilution (TCID50/mL)	Xpert EV	DAD^*b*^
5,000	3/3	3/3
1,000	3/3	3/3
500	3/3	3/3
250	3/3	3/3
100	3/3	3/3
50	1/3	3/3
25	0/3	3/3
Estimated LoD^*a*^ (TCID50/mL)	100	25

^*a*^ LoD, limit of detection.

^*b*^ DAD, direct disc.

### Inhibition assessment

Inhibition assay results are listed in Table 5. For the DAD method, qualitative inhibition was noted at a 1:5 dilution of blood in CSF. Since this method uses a direct sample without an extraction method, these results can be expected. For the UD method, qualitative inhibition was not noted for all tested levels of blood. This is likely due to the additional extraction step that precedes PCR.

**TABLE 5:** Results of the inhibition assay for DAD and UD methods

Diasorin DAD^*d*^				Diasorin UD^*e*^			
Preparation	Qualitative result	Ct	Ct change^*b*^	Preparation	Qualitative result	Ct	Ct change^*b*^
^*a*^Neat CSF	Detected	33.5	NA^*c*^	^*a*^Neat CSF	Detected	31.2	NA
Dilution 1:20	Detected	36.4	2.9	Dilution 1:20	Detected	31.6	0.4
Dilution 1:10	Detected	37.1	3.6	Dilution 1:10	Detected	32.2	1.0
Dilution 1:5	Not detected	0	NA	Dilution 1:5	Detected	33.2	2.0

^*a*^ Neat, pooled previously enterovirus positive clinical specimens.

^*b*^ Ct change, Ct (obtained) - Ct (without blood).

^*c*^ NA, not applicable.

^*d*^ DAD, direct disc.

^*e*^ UD, universal disc.

## DISCUSSION

While the Cepheid Xpert EV has been the standard of care for dedicated Enterovirus testing, its discontinuation creates a clinical need for rapid enterovirus testing. Herein, we describe two alternative methodologies with similar clinical performance. When weighing these alternatives, it is important to consider the testing time and ease of use. The standard of care Xpert EV assay requires minimal hands-on time, while the proposed DAD and UD methods utilize more technical input. The RNA extraction step necessary for the UD method requires a separate instrument and a more complicated workflow, which can add at least 1 hour of additional testing time.

In comparison to published studies, the proposed DAD and UD methods show similar clinical performance to enterovirus detection methods for CSF. The Xpert EV has a reported sensitivity of 96%–100% for enterovirus detection in CSF (9, 10). The FilmArray ME and QIAstat-DX ME panels have reported sensitivity of 89% and 88%–99% for enterovirus detection in CSF, respectively (6–8). Herein, we found direct positive percent agreement between the Xpert EV and the DAD and UD methods to be 79.6% and 86.4%, respectively. Based on this agreement and composite reference analysis, the clinical accuracy of the DAD method may be acceptable for clinical use in the microbiology laboratory.

A key strength of our study is the identification of two potential novel methods for the rapid diagnosis of enteroviral meningitis. The DAD method is a sample-to-answer assay, like the previously utilized standard of care Xpert EV method, which is an attractive option for large, busy microbiology laboratories. The UD method performs similarly, though it may have an increased analytical sensitivity. One must evaluate whether the extended RNA extraction time necessitated by the UD method justifies its application within their laboratory and for their patient population. Further clinical comparisons between these two methodologies are likely needed in future studies. CSF specimens can be contaminated with blood through the process of lumbar puncture. We show that blood contamination at a dilution of at least 1:5 can cause a lack of detection of enterovirus in CSF specimens using the DAD assay. The UD method did not show any decrease in sensitivity with possible blood contamination at the dilutions tested. This is likely due to the offline extraction step used for the UD method increasing sensitivity. Other studies utilizing the DAD have had success with diluting patient samples 1:1 in phosphate buffered saline to offset contamination, although this must be validated (11, 12). Overall, the results of this study and others indicate that for specimens with high blood inhibition, dilution can be validated, or alternatively, the UD method may be a more suitable choice for analyzing bloody CSF specimens.

One of the limitations of our study was the relative scarcity of acceptable positive specimens. For this study, 1 mL of remnant CSF was required; however, CSF is typically obtained in limited quantities. Consequently, some of the specimens tested were as old as 19 years past original collection, potentially introducing a selection bias, as the positive cohort comprised older specimens than the negative cohort. Additionally, extended frozen storage of RNA virus particles may have resulted in degradation, which could account for some of the discrepant results. Furthermore, the age of the specimens contributed to certain data collection challenges, rendering the demographic statistics incomplete.

Another potential limitation of our study is the absence of serotyping for the samples. Given the reported higher limit of detection for enterovirus D68 compared to other enteroviruses, the Xpert EV method may have failed to detect low viral loads of the D68 strain in the positive cohort. Without serotyping the positive cohort, definitive conclusions about the performance of the three assays for specific enteroviral strains in clinical samples cannot be drawn. We investigated the performance of the DAD assay against the Xpert EV assay for the detection of enterovirus D68 in non-clinical specimens, and the DAD proved to be analytically sensitive for the detection of this specific serotype.

In regard to analytical specificity, we have shown that the DAD and UD assays have high specificity for enterovirus detection among other common CSF pathogens. However, specificity was not assessed for rhinoviral infections, which represents a limitation of this study given known cross-reactivity between enterovirus and rhinovirus via nucleic acid testing methodologies. Although rhinoviral infections are exceedingly rare causes of meningoencephalitis, we acknowledge that the presence of rhinoviral nucleic acid has been shown to elicit cross-reactivity in nucleic acid amplification testing due to their degree of homology (13, 14). The Diasorin enteroviral primer pair is not known to have binding for rhinovirus; however, it targets the 5′ non-coding region of enteroviruses, so further validation for rhinovirus positive clinical samples is needed.

A rapid diagnostic test for diagnosing enteroviral meningitis is crucial to help guide treatment decisions and length of stay. With a relative lack of commercially available assays, we describe two potential alternatives for rapid diagnosis of enteroviral infections of CSF. Both the DAD and UD methods utilizing Diasorin analyte-specific reagents demonstrate similar clinical sensitivity compared to the previously available FDA-approved Xpert EV assay. The sample-to-answer DAD assay meets our laboratory’s validation criteria for acceptability and has been implemented for clinical use.

## SUPPLEMENTAL MATERIAL

Supplemental tables jcm.00864-25-s0001.docx Tables S1 and S2.

ASM does not own the copyrights to Supplemental Material that may be linked to, or accessed through, an article. The authors have granted ASM a non-exclusive, world-wide license to publish the Supplemental Material files. Please contact the corresponding author directly for reuse.
