# Supplementary material for: Evaluation of a sample-to-answer real-time PCR assay for enterovirus detection in cerebrospinal fluid
Source: J Clin Microbiol. 2025 Oct 21;63(11):e00864-25. doi: 10.1128/jcm.00864-25 (PMC12607652; doi:10.1128/jcm.00864-25)
Supplement: Supplemental tables — Tables S1 and S2. [file jcm.00864-25-s0001.docx]

**Supplemental Table 1.** Protocol definitions for direct amplification disc (DAD) optimization.

| **Protocol 1** |  |  |  |  |
| --- | --- | --- | --- | --- |
| **Step** | **Temperature (⁰C)** | **Time (seconds)** | **Ramp rate (⁰C /sec)** | **No. cycles** |
| *First cycle* | 75 | 180 | 5 | 1 |
| Mixing | 50 | 120 | - | 1 |
| Reverse transcription | 50 | 600 | 5 | 1 |
| *Second cycle* | 97 | 120 | 5 | 40 |
| Denature | 97 | 10 | 5 |  |
| Anneal | 56 | 10 | 5 |  |
| **Total run time: 66 minutes** |  |  |  |  |
| **Protocol 2** |  |  |  |  |
| **Step** | **Temperature (⁰C)** | **Time (seconds)** | **Ramp rate (⁰C /sec)** | **No. cycles** |
| *First cycle* | 75 | 120 | 5 | 1 |
| Mixing | 48 | 120 | - | 1 |
| Reverse transcription | 48 | 500 | 5 | 1 |
| *Second cycle* | 97 | 120 | 5 | 40 |
| Denature | 97 | 7 | 5 |  |
| Anneal | 56 | 30 | 5 |  |
| **Total run time: 74 minutes** |  |  |  |  |
| **Protocol 3** |  |  |  |  |
| **Step** | **Temperature (⁰C)** | **Time (seconds)** | **Ramp rate (⁰C /sec)** | **No. cycles** |
| *First cycle* | 75 | 180 | 5 | 1 |
| Mixing | 50 | 120 | - | 1 |
| Reverse transcription | 50 | 600 | 5 | 1 |
| *Second cycle* | 97 | 120 | 5 | 42 |
| Denature | 97 | 10 | 5 |  |
| Anneal | 56 | 10 | 5 |  |
| **Total run time: 68 minutes** |  |  |  |  |
| **Protocol 4** |  |  |  |  |
| **Step** | **Temperature (⁰C)** | **Time (seconds)** | **Ramp rate (⁰C /sec)** | **No. cycles** |
| *First cycle* | 75 | 180 | 5 | 1 |
| Mixing | 50 | 120 | - | 1 |
| Reverse transcription | 50 | 600 | 5 | 1 |
| *Second cycle* | 97 | 120 | 5 | 42 |
| Denature | 97 | 10 | 5 |  |
| Anneal | 56 | 10 | 5 |  |
| Extend | 58 | 30 | 5 |  |
| **Total run time: 88 minutes** |  |  |  |  |

**Supplemental Table 2.** Direct amplification disc (DAD) protocol optimization. *NA denotes a result beyond target cycle thresholds (>40)

| **Protocol 1**  Nucleic acid amount (copies/ mL) |  |  |  |  |
| --- | --- | --- | --- | --- |
|  | *With RNasin* | *With RNasin* | *Without RNasin* | *Without RNasin* |
|  | Enterovirus cycle threshold | Internal control cycle threshold | Enterovirus cycle threshold | Internal control cycle threshold |
| 100,000 | 31.7 | NA | 34.0 | NA |
| 10,000 | 35.1 | 38.9 | 37.3 | NA |
| 1,000 | 38.8 | NA | NA | NA |
| 500 | 39.1 | 39.7 | NA | NA |
| **Protocol 2**  Nucleic acid amount (copies/ mL) |  |  |  |  |
|  | *With RNasin* | *With RNasin* | *Without RNasin* | *Without RNasin* |
|  | Enterovirus cycle threshold | Internal control cycle threshold | Enterovirus cycle threshold | Internal control cycle threshold |
| 100,000 | 31.6 | 34.6 | 34.2 | 38.3 |
| 10,000 | 35.4 | 34.6 | NA | NA |
| 1,000 | NA | 35.3 | NA | NA |
| 500 | 38.0 | 35.2 | NA | NA |
| **Protocol 3**  Nucleic acid amount (copies/ mL) |  |  |  |  |
|  | *With RNasin* | *With RNasin* |  |  |
|  | Enterovirus cycle threshold | Internal control cycle threshold |  |  |
| 100,000 | 32.1 | 36 |  |  |
| 10,000 | 34.8 | 35.3 |  |  |
| 5,000 | 36.4 | 35.9 |  |  |
| 1,000 | 38.3 | 35.3 |  |  |
| 500 | 39.4 | 35.6 |  |  |
| 250 | NA | 35.3 |  |  |
| 125 | NA | 35.3 |  |  |
| 62.5 | NA | 35.3 |  |  |
| **Protocol 3 with filtration step**  Nucleic acid amount (copies/ mL) |  |  |  |  |
|  | *With filtration column* | *With filtration column* | *Without filtration column* | *Without filtration column* |
|  | Enterovirus cycle threshold | Internal control cycle threshold | Enterovirus cycle threshold | Internal control cycle threshold |
| 5,000 | 35.2 | 37.0 | 36.2 | 36.4 |
| 500 | NA | 37.6 | 40.0 | 37.1 |
| 250 | 41.7 | 39.2 | 40.3 | 38.0 |
| 125 | NA | 37.1 | NA | 36.8 |
| **Protocol 4**  Nucleic acid amount (copies/ mL) |  |  |  |  |
|  | *With RNasin* | *With RNasin* |  |  |
|  | Enterovirus cycle threshold | Internal control cycle threshold |  |  |
| 100,000 | 36.6 | 33.8 |  |  |
| 10,000 | 34.5 | 33.6 |  |  |
| 5,000 | 36.3 | 34.6 |  |  |
| 1,000 | 37.3 | 34.5 |  |  |
| 500 | 37.2 | 33.9 |  |  |
| 250 | 40.0 | 33.7 |  |  |
| 125 | NA | 34.1 |  |  |
| 62.5 | NA | 34.6 |  |  |
| **Protocol 4 with filtration step**  Nucleic acid amount (copies/ mL) |  |  |  |  |
|  | *With filtration column* | *With filtration column* | *Without filtration column* | *Without filtration column* |
|  | Enterovirus cycle threshold | Internal control cycle threshold | Enterovirus cycle threshold | Internal control cycle threshold |
| 5,000 | 35.6 | 32.3 | 36.7 | 32.0 |
| 500 | NA | 32.2 | NA | 33.0 |
| 250 | NA | 32.1 | NA | 32.7 |
| 125 | NA | 32.0 | 39.9 | 32.9 |
